# Musculoskeletal complaints and its economic impact in an Iranian army hospital

**DOI:** 10.1186/s12891-024-07511-0

**Published:** 2024-05-18

**Authors:** Soosan G Soroosh, Abolfazl Farbod

**Affiliations:** 1 Rheumatology Department, AJA University of Medical Sciences, Tehran, Iran; 2 Headache Department, Iranian Center of Neurological Research, Neuroscience Institute, Tehran University of Medical Sciences, Tehran, Iran

**Keywords:** Musculoskeletal, Cost, Military, Army

## Abstract

**Aim:**

Musculoskeletal conditions constitute a remarkable portion of disability cases in the military. This study evaluated the distribution and types of musculoskeletal problems and estimated the direct and indirect costs due to these complaints in an Iranian military hospital.

**Methods:**

All medical records of patients with musculoskeletal complaints that were referred to the medical committee of a military hospital, including rheumatology, orthopedics, and neuro-surgical specialists, from 2014 to 2016, were reviewed. Details of each complaint and the final opinion of the medical committees were recorded. The cost of each diagnostic step was calculated based on the recorded data. The treatment costs were estimated for each complaint by calculating the average cost of treatment plans suggested by two specialists, a physical medicine and a rheumatologist. The estimated cost for each part is calculated based on the army insurance low. Indirect costs due to absences, inability to work, and disability were assessed and added to the above-mentioned direct costs. Statistical analysis was performed using SPSS version 21.

**Results:**

2,116 medical records of the committee were reviewed. 1252 (59.16%) cases were soldiers (who had to spend two years of mandatory duty in the army), and 864 (40.83%) cases were non-soldiers. The three most common complaints were fractures (301 cases, 14.22%), low back pain due to lumbar disc bulges and herniations (303 cases, 14.31%), and genu varus/genu valgus (257 cases, 12.14%). The most affected sites were the lower limbs and vertebral column. According to an official document in these subjects’ records, 4120 person-days absent from work were estimated annually, and nearly $1,172,149 of annual economic impact was calculated.

**Conclusion:**

Musculoskeletal problems are common in the army, and establishing preventive strategies for these conditions is essential. The conservative and medical approach and the proper education for correct movement and the situation should be mentioned for the reduction of disability and its economic burden on the army’s staff.

## Background

Musculoskeletal problems pose a remarkable impact on militaries around the world. This high burden in different aspects arises from the combination of the physical nature of military activities, its high-demanding physical jobs, and the types of working environments in the military. Musculoskeletal diseases result in disability and lost duty days or absence. Decreased productivity and function, states of chronic pain, burnout in personnel, long-term disability, and its financial impacts are associated with musculoskeletal disorder’s economic burden [1–3]. It was revealed that around 50% of soldiers are injured during military training [4–6]. From 70 to 96% of these injuries are musculoskeletal [7–10]. In the US, the cost of musculoskeletal injuries in Air Force basic military trainees during three years reached over 43 million US dollars [9]. These numbers and figures reflect the importance of musculoskeletal disorder conditions in military soldiers and personnel. Although these injuries are highly prevalent among military recruits and personnel, there is a remarkable lack of studies on the epidemiology of these injuries around the world.

and particularly in Iran.

In Iran, all 18-year-old males should enter the military training program, which is associated with a relatively high frequency of musculoskeletal injuries due to intense training programs and physical demands of soldiers in military programs and duties. Military personnel and soldiers with any medical disorder or abnormality that makes them totally or partially unfit for active duty are referred to army medical committees in military hospitals. In these committees, the medical complaints of these patients are assessed. The orders verdict by medical committees determines the future status of the patient in his job (for example, early resignation from military service, reduction in duty hours per day, release from military service, etc.). Rheumatology, neurosurgery, and orthopedics evaluated these musculoskeletal complaints in this committee according to their complaints.

To design and achieve a preventive strategy for reducing the incidence and burden of musculoskeletal problems in the army, it is necessary to obtain a scheme for musculoskeletal complaints. Another critical factor in managing health systems is the allocation of financial resources. Proper allocation of financial resources leads to a better health profile of patients under a system. Estimating direct and indirect costs due to musculoskeletal complaints is the first step in revising the financial resource allocation. For this purpose, this study was conducted to evaluate the distribution and types of musculoskeletal conditions and then to estimate direct and indirect costs due to these complaints in medical committees of an Iranian army hospital.

## Methods

This retrospective study was conducted at a military hospital in Tehran, Iran. The study involved retrieving and reviewing all medical records from three Army medical board committees (specifically rheumatology, neurosurgery, and orthopedics) at the Army hospital in Tehran, Iran, from 2014 to 2016. The patients’ complaints were evaluated by the rheumatology, neurosurgery, and orthopedics committees at the Army Hospital, and if their complaints were endorsed, it led to adjustments in their duty schedules. These committees comprised rheumatologists, orthopedic surgeons, and neurosurgeons, and the decision-making was founded based on their expert opinions and army insurance references. This study included all medical records of each musculoskeletal disorder that interrupt the proper duty, such as release from military service, based on the expert opinions of the specialists mentioned above. Demographic data (age, gender, military service duration), chief complaint, diagnosis, committee’s order, involved anatomical location, and related findings from imaging were recorded. Patients were classified as soldiers and non-soldiers. The soldier was defined as an unemployed, non-official person who should have a 2-year obligatory duty. The soldiers I am referring to are the ones who are in mandatory military service(conscripts) or in other words, are conscripted into the army and not employed by the army in Iran.

while a non-soldier was described as an employed, official military staff. Orders differed for soldiers and non-soldiers. Orders for soldiers included permanent and temporary releases from military service. The level of disability is categorized as class I to IV of disability. In disability class, I referred to patients with mild illness, injury, or disability who can continue their duties as usual. Disability class II relates to patients with moderate disease, injury, or disability, and they have reduced daily duty time during normal non-combat and non-operational conditions. Disability class III refers to patients whose illness, injury, or disability is so severe that they benefit from service discharge in all standard, combat, and operational conditions. Disability class IV refers to patients whose permanent illness, injury, or disability results in a loss of efficiency to the extent that they cannot continue serving in any armed forces units. Patients with class IV were considered candidates for early retirement from military service. Class III and IV patients were given several duty-free hours per day according to the severity of their disability.

The study’s tricky part was calculating and estimating the economic burden of these complaints. We faced two drawbacks in the financial analysis process; the first was the nature of the medical committee, which is mainly a diagnostic unit in the hospital, and its records usually do not cover the therapeutic steps afterward. The second was that some information about military personnel was confidential, and we could not access them. So, we designed a method to estimate the different costs of these patients. We extracted the diagnostic steps for the patients from the committee’s records and calculated the prices of procedures, imaging, visits, etc., according to the available official army insurance fees. Then, we asked two specialists, including physical medicine and rheumatologists, to estimate the cost of diagnostic and treatment plans for each complaint. If there were disagreements regarding their diagnosis and treatment plans, they resolved them through consensus reached via discussion. We calculated the cost of each complaint’s presumed diagnostic and treatment plan. To estimate the indirect costs of the hospital, a study was recently conducted in an Iranian army hospital [11]. Based on a cost analysis, this study has conclusively determined that 48% of the estimated disease-related expenses in a hospital are attributable to indirect costs, including expenses for medication, space depreciation, human resources, etc. We calculated this augmentation and added it to the previously estimated costs. In the next step, duty-free hours for class III and IV patients were summed up and converted to the number of duty-free days (each 8 h was considered one duty-free day). The estimation of work-off days in the musculoskeletal complaints was based on the medical opinion committee for each diagnosis. The analysis of financial loss due to work-off days was based on the average salary paid by the government. In Iran, the 2-year military service is obligatory for each man aged over 18 years old. However, Iranian men can be released from obligatory military service under several circumstances, such as medical conditions (severe physical disabilities that significantly impair the individual’s ability to perform military duties, chronic health conditions that require ongoing medical treatment and would pose a significant challenge in an army environment, etc.). In this study, we utilized expert opinions to estimate the cost of the soldier’s release due to medical conditions from the committees of rheumatology, neurosurgery, and orthopedics. While the precise salaries of the armed forces could not be accessed, data from official statistics in 2016 facilitated an estimation of the average wage. Additionally, The reference for health fees and costs was derived from Iran’s Army Health insurance fee for each diagnostic test or treatment. Person-day was calculated by multiplying the number of a person’s absence by the number of days. In contrast, person-month is calculated by multiplying the number of persons absent by the number of months. Finally, over three years of retrospective data collection and based on the health disability classification for each patient in every committee, the total absenteeism was computed. Statistical analysis was performed using SPSS version 21. Confidentiality of the data was respected during all the study steps.

## Results

Overall, 2116 patients with a mean age of 25.84 ± 8.17 years were identified. 1252 (59.16%) and 864 (40.83%) of the records were related to soldiers and non-soldiers, respectively. Two thousand seventy patients (97.82%) were male, and 46 (2.17%) of the remaining patients were female. The most common conditions in our study included fractures (301 cases, 14.22%), lumbar bulged disc and hernia (303 cases, 14.31%), genu varum and genu valgum (257 cases, 12.14%), flat feet (209 cases, 9.87%), meniscus tear (176 cases, 8.31%), lumbar degenerative changes (145 cases, 6.85%), cervical bulged disc and hernia (70 cases, 3.30%), tear of knee ligaments (53 cases, 2.50%) and lumbar spondylolysis (45 cases, 2.12%).

### Rheumatology committee

Twenty-seven cases were present. Five patients (18.51%) were soldiers, and 22 patients (81.48%) were non- soldiers. Five patients (18.51%) were female, and 22 (81.41%) were male. The mean age of patients was 38.11 ± 8.14 years. The duty-free hours were 16.47 h per day, leading to a figure of 25.04 person-months. Total lost work days were estimated to be around 225.8 person-days during the study.

For soldiers, one patient (7.40%) was free of combat, and three patients (11.11%) were permanently released from military service. Common diagnoses in this category are listed in Table 1. Affected sites included the spine (5 cases, 18.51%), wrist (3 cases, 11.11%), femur (2 cases, 7.40%), pelvis (two cases, 7.40%), peripheral joints (one case, 3.70%) and foot (one case, 3.70%).

**Table 1:** The frequency of musculoskeletal diseases stratified based on different committees among soldiers and non-soldier military personnel

Committees	Soldiers	Frequency, *n* (%)	Non-Soldiers	Frequency, *n* (%)
*Rheumatology committee* *(5 soldiers and 22 non-soldiers)*	Gout	2 (40%)	Osteopenia and osteoporosis	8 (36.36%)
	Ankylosing spondylitis	2 (40%)	Seronegative arthropathies	6 (27.27%)
	Osteoporosis	1 (20%)	Osteoarthritis and arthrosis	3 (13.63%)
*Neurosurgery committee* *(387 soldiers and 418 non-soldiers)*	Lumbar hernia and bulged disc	131 (33.85%)	Lumbar hernia and bulged disc	172 (41.14%)
	Lumbar degenrative changes	53 (13.69%)	Lumbar degenrative changes	92 (22%)
	Cervical hernia and bulged disc	43 (11.11%)	Cervical degeneration	35 (8.37%)
	Lumbar spondylolysis	18 (4.65%)	Cervical hernia and bulged disc	27 (6.45%)
	Radiculopathy	15 (3.87%)	Lumbar spondylolysis	27 (6.45%)
	Spondylolisthesis	13 (3.35%)	Lumbar spinal stenosis	21 (5.02%)
	Lumbar spinal stenosis	13 (3.35%)	Radiculopathy	20 (4.78%)
	Traumatic vertebral fracture	10 (2.58%)	Spondylolisthesis	15 (3.58%)
	Cervical degeneration	10 (2.58%)	Traumatic vertebral fracture	14 (3.34%)
	Kyphoscoliosis	9 (2.32%)	History of lumbar fusion	**7** (1.67%)
*Orthopedics committee* *(860 soldiers and 424 non-soldiers)*	Genu varum/ genu valgum	215 (25%)	Fractures	133 (31.36%)
	Flat foot	193 (22.44%)	Meniscus tear	68 (16.03%)
	Fractures	168 (19.53%)	Genuvarum/genuvalgum	42 (9.90%)
	Meniscus tear	108 (12.55%)	Limbs’ vascular injury	30 (7.07%)
	Knee ligaments tear	37 (4.30%)	Patellar involvements	25 (5.89%)
	Joints’ range of movement limitation	17 (1.97%)	Knee ligaments tear	24 (5.66%)
	Limbs’ vascular injury	16 (1.86%)	Joints’ recurrent or old subluxation	22 (5.18%)

### Neurosurgery committee

Eight hundred-five cases were found. Three hundred eighty-seven patients (48.07%) were soldiers, and 418 patients (51.92%) were non-soldiers. Fifteen patients (1.86%) were female, and 790 (98.13%) were male. The mean age of patients was 29.81 ± 7.49 years. Total duty-free hours were 193.2 h per day, leading to 293.82 person-months. Entire lost work days were estimated to be around 6099.2 person-days.

For soldiers, 179 patients (22.23%) were combat-free, and 180 patients (22.36%) were permanently released from military service. Common diagnoses in this category are shown in Table 1. The most common affected sites included L5-S1 (173 cases, 21.49%), L4-L5 (153 cases, 19.00%), C5-C6 (38 cases, 4.72%), L3-L4 (54 cases, 6.70%) and C6-C7 (19 cases, 2.36).

### Orthopedics committee

One thousand two hundred eighty-four cases were identified. Eight hundred sixty patients (66.97%) were soldiers, and 424 patients (33.02%) were non-soldiers. Twenty-six patients (2.02%) were female, and 1258 (97.97%) were male. The mean age of patients was 23.08 ± 7.14 years. Duty-free hours were 295.32 h per day, leading to 449.13 person-months. Total lost work days was estimated to be around 6037.8 person-days.

For soldiers, 694 patients (54.04%) were combat-free, and 91 patients (7.08%) were permanently released from military service. Common diagnoses in this category are listed in Table 1. Lower limbs were the most affected locations, including knee in 534 cases (41.58%), foot and leg in 396 cases (30.84%), long bones in 116 cases (9.03%), and hip in 49 cases (3.81%). Upper limbs were involved in 154 cases (11.99%). The back, trunk, and neck comprised 35 cases (2.72%) of the complaints.

### Overall cost

In total, the estimated cost of musculoskeletal diseases was 5,713,010,400 tomans (equal to 3,907,164.16 US dollars), which was calculated as 1,713,903,120 tomans per year (equivalent to 1,172,149.77 US dollars). Further details are demonstrated in Table 2.

**Table 2:** The estimated annual economic burden of musculoskeletal diseases in different committees (in US dollars, adjusted for 25 December 2023)

Committee	Costs of illness	Duty-free hours salary	Work-off days salary	Soldiers’ release cost	Total
Rheumatology	1827.70	15133.68	4548.97	1541.77	23052.12
Neurosurgery	144380.25	177581.95	120592.63	92507.12	535061.96
Orthopedics	174183.99	271446.69	121637.52	46767.50	614035.69
Total	320391.94	464162.31	246779.12	140816.40	1172149.77

## Discussion

As we reviewed 2116 medical records of soldiers and non-soldiers in an Iranian military hospital, we noticed that most complaints in our patients arise from involvements in lower limbs (mainly knee and foot) and spine (primarily disc disorders). About these two regions, the upper limbs and trunk were far less involved. Other studies have revealed results similar to our findings. Nye et al. [9] have reported that in recruits of military training in the US Air Force, 78.4% of injuries were related to lower limbs, while upper limbs were affected in 7.7% of cases. Spinal involvement occurred in 6.2% of patients. A study by Mehri-Najafi et al. [10] on formal Iranian military personnel showed that musculoskeletal complaints constitute 96.2% of involvements during military training programs, and the most common site was feet and ankles (27.6%).

There was a remarkable difference regarding complaints in the orthopedics committee between soldiers and non-soldiers; as in soldiers, nearly half of the complaints were related to genu varum/genu valgum and flat feet, which are the two most common complaints in soldiers to be released from a military training program but in non-soldiers, the pattern differs. Non-soldiers have passed the military training phase, so the rate of these complaints has significantly decreased and replaced with fractures and meniscus tears. In the neurosurgery committee, the pattern of complaints does not differ between soldiers and non-soldiers because disc disorders and degenerative changes are prevalent in all age groups and are far more prevalent in comparison to other neurosurgical conditions.

Musculoskeletal complaints in our study were associated with a total cost of 1,172,149.77 US dollars per year. Due to the lack of studies in the literature similar to our survey regarding the source of evaluation in the military medical committees and the absence of official numbers and figures on the burden of musculoskeletal disorders in Iran, we could not compare our findings to other Iranian studies. Still, some studies have been conducted in our parts of the world, which can be mentioned here. Anderson et al. in 1993 [12] evaluated the cost of rheumatologic disorders and musculoskeletal complaints in the US Air Force and reported it to be around 2.7 million dollars per year. Nye et al. [9] investigated the cost of recruits’ military training in the US Army during a 3-year period, which led to the findings of 8.7 million dollars in medical expenses and 35 million dollars in non-medical costs. Launder et al. [13] found that, on average, 29,435 days were lost annually in the army due to sports injuries, equal to 13 and 11 absent work days for men and women, respectively.

The combination and nature of common musculoskeletal injuries and complaints in our study indicate that these problems are largely preventable, so it is rational that new strategies in training soldiers and planning exercises should be undertaken to reduce economic costs and physical injuries. Previous research supported this idea, indicating lower risk of musculoskeletal injuries by resistance training (relative risk = 0.82; 95%CI: 0.72, 0.93), lower risk of acute ankle injuries by neuromuscular and balance training (close risk = 0.34; 95%CI: 0.15, 0.75), lower risk of anterior knee pain by Anterior Knee Pain Prevention Training Program (relative risk = 0.27; 95%CI: 0.14, 0.54), and lower risk of medial tibial stress syndrome by supervised gait retraining program in combination with neuromuscular and strengthening exercises (relative risk = 0.25; 95%CI: 0.05, 0.53) among military personnel [14].

Additionally, our study on musculoskeletal disorders in an Iranian military hospital has significant implications for resource allocation, financial planning, policy development, healthcare management, occupational health and safety, preventative strategies, and cost-benefit analysis within the military healthcare system.

Our study had some limitations. The nature of documents in the records of medical commissions of military hospitals are mainly diagnostic and do not contain information on following treatment steps. In addition, simple and routine complaints such as sprains, strains, and non-specific pain of limbs are not usually presented to the medical commission, but they constitute a high percentage of musculoskeletal complaints in clinics. It is important to note that the records under review encompassed a spectrum of conditions, including injuries and old sequels, chronic problems, congenital deformities, and similar. This inclusive approach consistently identifies and estimates all complaints related to musculoskeletal disorders evaluated by the Army hospital’s medical board committee and led to changes in the duty schedule of soldiers and military staff. The studies in the literature are mainly focused on injuries, which does not allow us to compare our research findings with those of others.

The findings of this study are directly applicable to Iranian military healthcare settings. Still, they may have limited generalizability to civilian populations or military contexts in other countries due to potential variations in demographics, occupational exposures, disease patterns, and cost structures. Further research in diverse military contexts can enhance the broader applicability of the findings.

## Conclusion

Musculoskeletal complaints are common in the army and cause high economic costs for the military. So, the establishment of prevention strategies for these conditions is essential.

## Data Availability

The datasets used and analyzed during the current study are available from the corresponding author upon reasonable request.

## References

[1] Jones BH, Cowan DN, Tomlinson JP, Robinson JR, Polly DW, Frykman PN (1993). Epidemiology of injuries associated with physical training among young men in the army. Med Sci Sports Exerc.

[2] Davidson PL, Chalmers DJ, Wilson BD, McBride D (2008). Lower limb injuries in New Zealand Defence Force personnel: descriptive epidemiology. Aust N Z J Public Health.

[3] Jones BH, Thacker SB, Gilchrist J, Kimsey CD, Sosin DM (2002). Prevention of lower extremity stress fractures in athletes and soldiers: a systematic review. Epidemiol Rev.

[4] Almeida SA (1999). Epidemiological patterns of musculoskeletal injuries and physical training. Med Sci Sports Exerc.

[5] Napik JJ, Graham B, Cobbs J, Thompson D, Steelman R, Jones B (2013). A prospective investigation of injury incidence and injury risk factors among army recruits in military police training. BMC Musculoskelet Disord.

[6] Sharma J, Greeves JP, Byers M, Bennett AN, Spears IR (2015). Musculoskeletal injuries in British Army recruits: a prospective study of diagnosis-specific incidence and rehabilitation times. BMC Musculoskelet Disord.

[7] Lovalekar M, Abt JP, Sell TC (2017). Accuracy of recall of musculoskeletal injuries in elite military personnel: a cross-sectional study. BMJ Open.

[8] Hua W, Chen Q, Wan M (2018). The incidence of military training-related injuries in Chinese new recruits: a systematic review and meta-analysis. J R Army Med Corps.

[9] Nye NS, Pawlak MT, Webber BJ, Tchandja JN, Milner MR (2016). Description and rate of Musculoskeletal Injuries in Air Force Basic Military trainees, 2012–2014. J Athl Train.

[10] Najafi Mehri S, Sadeghian M, Tayyebi A, Karimi Zarchi AA, Asgari AR (2010). Epidemiology of physical injuries resulted from military training course. J Mil Med.

[11] Noori m, Markazi Moghaddam N, Goudarzi R, Meshkani Z (2016). Surveying activity based costing of final units (a case study in one of the Armed. Forces Hospitals) Jhosp.

[12] Anderson ST, Charlesworth RW (1993). Rheumatologic disease among Air Force recruits: a multimillion-dollar epidemic. Semin Arthritis Rheum.

[13] Lauder TD, Baker SP, Smith GS, Lincoln AE (2000). Sports and physical training injury hospitalizations in the army. Am J Prev Med.

[14] Dijksma I, Arslan IG, van Etten-Jamaludin FS, Elbers RG, Lucas C, Stuiver MM (2014). Exercise Programs to reduce the risk of Musculoskeletal Injuries in Military personnel: a systematic review and Meta‐analysis. PM&R.

